# Alloantigenic recognition properties of CD8^+^ regulatory T cells

**DOI:** 10.1186/1479-5876-9-S2-P29

**Published:** 2011-11-23

**Authors:** Elodie Picarda, Ignacio Anegon, Carole Guillonneau

**Affiliations:** 1 INSERM UMR643 – IUN, Nantes, France

## Background

We recently reported that in a rat major histocompatibility complex (MHC) mismatched heart allograft model, treatment with CD40Ig, a chimeric molecule that blocks CD40L, leads to indefinite allograft survival mediated by CD8^+^CD45RC^low^ Tregs [1]. Although essential, the exact role of TCR/MHC/peptide interaction in Treg activity is still unknown. We therefore characterize the allogeneic peptide(s) recognized and the TCR usage of the CD8^+^CD45RC^low^ Tregs.

## Material and methods

Allogeneic peptide(s) were derived from polymorphic regions of donor MHC molecules [2,3]. Sixty-two overlapping peptides of sixteen amino acids (aa) were tested in a coculture of Tregs with syngeneic pDCs (ratio 4:1). Moreover, the repertoire of the TCR of the CD8^+^ Tregs was studied by flow cytometry analysis and sequencing the CDR3 region.

## Results

After six days of culture, two peptides in particular led to the activation of Tregs, as shown by the upregulation of the CD25 molecule (from 25.89% to 29.27% of CD25 expression). These activator peptides were characterized by prominent amino acids (aa) at rather central position, which could result in a large TCR repertoire diversity of the specific Tregs. We showed previously that CD8^+^CD45RC^low^ Tregs expressed a specific altered V 11 repertoire, with the same CDR3 length in all animals (9 aa). This upregulation was confirmed at the protein level, since 19.9±3.7% of Tregs from a CD40Ig-treated animal expressed the V 11 chain compared to 6.1±2.3% in naïve ones. Sequencing of 160 clones of V 11 TCRs from six long-surviving animals suggested a preferential use of a 9 aa long CDR3 and a particular J region (J 1.6). Interestingly, conserved sequences were frequently found but no common clonotype was shared between animals, suggesting the private nature of the repertoire.

## Conclusion

This study demonstrated that CD8^+^CD45RC^low^ Tregs recognize two potential allogeneic epitopes leading to a private TCR repertoire.
